# Attack Detection Using Network Coding in IoT Environment

**DOI:** 10.3390/s20041180

**Published:** 2020-02-21

**Authors:** Yong Lee, Goo Yeon Lee

**Affiliations:** 1 Independent Researcher, Chuncheon 24341, Korea; 2 Department of Computer and Communications Engineering, Kangwon National University, Chuncheon 24341, Korea

**Keywords:** Internet of Things, network coding, attack detection, recovery

## Abstract

Network coding is a reasonable way to increase network efficiency in response to an increase of sensed data in the Internet of Things (IoT). In network coding, intermediate nodes combine packets received from neighboring nodes, transform, and transmit encoded packets that can be decoded at the destination. This scheme is based on trust among nodes. If any malicious node joins the network, it can act as an intermediate node that could fabricate encoded packets. It might be more difficult to identify the authenticity of such encoded packets since packets that are received at the destination might not originate from a single source, but be combined with several other packets originating from multiple sources. In this paper, we propose a scheme on how to detect attacked packets among the received packets at a destination and how to recover the original message from the packets including the attacked *“look-like-valid”* packets. This scheme shows that a destination could recover the valid message with just the received packets including some attacked packets and will result in a quite efficient performance in network coding.

## 1. Introduction

With the provision of many applications based on Internet of Things (IoT), the amount of data processing in the network is rapidly increasing due to the increase in sensing information. Therefore, network coding is a good solution for improving network throughput and efficiency [1,2,3]. In network coding, an intermediate node combines packets that are received from neighboring nodes, then transforms them into encoded packets that can be decoded at destination [4,5]. This approach is particularly suited for IoT environments because it is primarily used in multi-hop multipath network architectures from source to sink to provide robustness and error tolerance for the network. In the IoT, wearable devices and sensor nodes collect, transmit, and relay data. Encoded packets are a combination of packets received from multiple sources, so that quantitatively combined information is transmitted as a result [1,3,4,5]. This method of mixing information and increasing the amount of information has the advantage of substantially increasing the transmission efficiency and allowing the network to be flexible in communication, hardware, or relay errors [1,3]. This scheme can only operate correctly if the network topology can be configured with trusted nodes. If a malicious node legitimately participates in the network configuration, it is possible to insert a forged encoded packet as an intermediate node. Since the packet received at the destination is not from one source but is a combination of packets from multiple sources, it is not easy to recognize whether the received packet is valid. Therefore, a network architecture using network coding has a high risk of information forgery by structurally malicious nodes.

In the IoT environment, the joining and identification of trusted nodes is a controversial topic, especially in the case of an autonomous network configuration by mobile nodes. Threats can be caused not only by the invading malicious nodes, but also by internal nodes that legally participate in the network configuration. In the case of threats by legitimate internal nodes, the attack will come from a trusted node, making it more difficult to determine the identity of the attacker. Cryptographic algorithms, such as digital signatures and encryption, can be applied to network coding, but if a malicious node that legitimately joins the network configuration deliberately manipulates, forges, and performs an internal attack with a legitimate digital signature or encryption, the other nodes will not be aware of it.

When the destination trusts the received packets, it performs a message recovery mechanism with those packets. Recovery by valid packets and recovery by packets disguised as valid packets by an internal attack will generate different messages. Let us consider an example. Assume that a node generates a message that is decomposed into *b* packets, transforms them into *n* packet combinations with redundancy, m(n=b+m). If all *n* encoded packets would arrive at a destination but any intermediate node performs an internal attack, these packets all appear to be valid, but may include *“look-like-valid”* but actually *“attacked”* packets. Since packets can have a valid digital signature or encryption form and the destination cannot verify whether the packet is valid by digital signature verification or decoding, it is an important issue how to authenticate the packets received at the destination.

In this paper, we propose a valid message identification method of network coding considering the IoT environment where IoT devices can be added freely. This method detects the presence of an attacked packet among the packets received by the destination and makes it possible to determine a valid message when packets recover several different messages due to attacked but *“look-like-valid”* packets. Therefore, the burden of retransmission due to the attack can be reduced. This scheme shows that with a high probability, the destination can recover a valid message with received packets without requiring retransmissions, which will give quite throughput improvement.

The paper is organized as follows. We consider the previous work of network coding security in Section 2. We describe the system model and operations of our scheme in Section 3. Section 4 investigates characteristics of the model and the detailed algorithm. Section 5 shows performance analysis and the results. We conclude in Section 6.

## 2. Related Work

This section discusses work related to the security of network coding in IoT environment. Types of attacks that can occur in network coding include the Byzantine attack, impersonations, and pollution attacks [6]. To date, a lot of work has been researched to prevent these attacks or to detect them in network coding security.

One of the areas that have been most focused on is applying a cryptographic algorithm and a digital signature scheme to identify pollution packets. Peralta et al. proposed a homomorphic cryptography model for network coding to enhance end-to-end security, such as ensuring the confidentiality of data in the Internet of Things [1]. Boneh et al. have proposed a homomorphic signature scheme which could prevent any attacks by arbitrary nodes in network, and they have insisted that the destination could use this signature scheme to filter out corrupted packets, and even intermediate nodes could discard corrupted packets with computational overhead [4].

Yu et al. proposed an XOR network coding security scheme that can filter pollution attacks in a few hops using probabilistic key pre-distribution and message authentication codes (MACs) [7]. Shafagh et al. noted that the security of data is important when service providers and users access the cloud because the cloud is used to store data collected by an IoT application. To solve this problem, they proposed and evaluated a data sharing algorithm that applies homogeneous encryption algorithms as a data protection platform in the cloud [8].

Li et al. applied a network coding signature scheme to guarantee the authenticity of data according to the feature that it is difficult to share the key in the IoT where it is easy to add nodes, and proposed a signature method for data collected by various devices using their own authentication key [9]. In addition, Wu et al. [10] proposed a method that verifies whether a packet is valid by applying a digital signature algorithm in case a network coding is used in a vehicular area network (VANET) and a vehicle cannot recover a message due to a pollution attack. Cheng et al. showed that multi-generation pollution attacks are possible when homogeneous subspace signature schemes are used to cope with pollution attacks in network coding and described an algorithm that solves this problem by improving the key distribution method [11].

Chen et al. proposed a method of applying error detection and error correction techniques to encoded packets. This paper showed that the throughput can be improved by allowing the intermediate nodes to correct the packet [12]. Mamidwar et al. analyzed studies to prevent the rapid spread of pollution attacks from network coding throughout the network. These studies consisted of error correction, localization of malicious nodes, and pollution packet detection [13]. Ayday et al. have worked a security service called a location aware network coding scheme that provide data authenticity through node collaboration and data redundancy in any environment where nodes are dense enough such that an event can be sensed by multiple nodes [14].

Wang et al. proposed a trust scheme of defining reliable nodes’ behavior in order to prevent pollution attacks from spreading rapidly to the network [15]. Ji et al. proposed a distributed detection algorithm to prevent wormhole attack by malicious nodes using expected transmission counts in the wireless network coding systems [16].

In the IoT, when a lot of data is stored in a data collection center, network coding may be applied to the data for retrieval efficiency or security of information. Oliveira et al. applied network coding to the storage of data and optimized the added redundancy to ensure reliable data storage and to retrieve more packets at minimal cost [17]. Lei et al. showed that the network coding can be efficiently applied between data producers and consumers to handle large amounts of data transmission in the named data networking (NDN) model to provide IoT applications [18].

Cebe et al. proposed a method of applying network coding to transmit sensing data using blockchain technology in the IoT environment. In the proposed scheme, blockchain technology has a large amount of computation and blocks, and the overhead of a long time delay is involved, whereas network coding technology transmits data packets by combining them, which helps to solve the problem of blockchain by increasing throughput [19].

Lima et al. have explained the security vulnerabilities of network coding and compared the differences between attack scenarios in network coding combined with classical cryptography [6].

Dong et al. have described the framework with a focus on network coding systems designed for wireless mesh networks, identified potential security vulnerabilities that could seriously degrade system performance, and defined security goals and challenges [20].

Zhao et al. have studied network coding using real-world BitTorrent measurements called NCTorrent on a wireless body area network (WBAN) for reliable medical data transfer, in which data are transferred via relays from multiple wireless body sensors to the monitoring stations. The study concluded with a pessimistic conclusion that network coding might not be beneficial for real-world BitTorrent systems [21].

## 3. System Model and Descriptions

**Figure 1:**
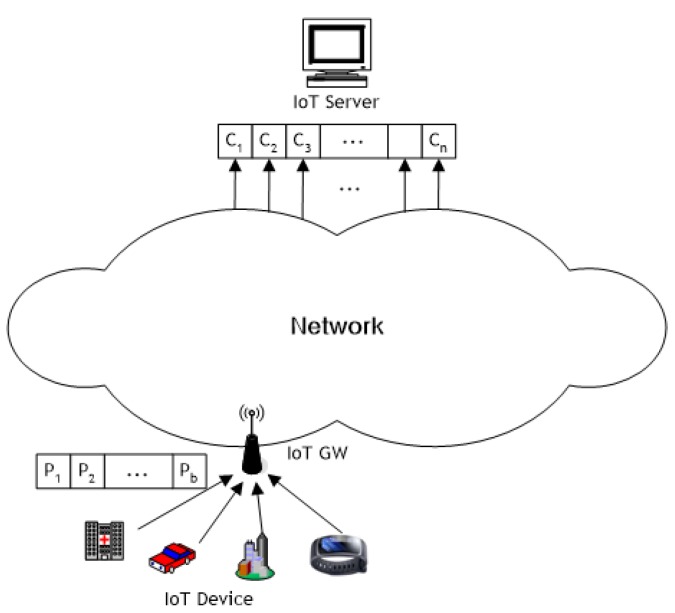
An example of network coding in an Internet of Things (IoT) network.

In this paper, we do not consider transmission errors. However, assuming errors in transmission, our scheme also applies in the case that error processing methods are applied. That means packets with some errors will be discarded at the destination by error detection methods which means no arrivals, and error corrected packets by error correction methods are considered as normal arrivals at the destination. In network system, a node transmits packets transformed by applying random linear combination to its received packets as random network coding. In this case, a message means data of any size generated by a source, and a packet means a fragment of a message divided into fixed size length for transmission. Figure 1 shows an example in which information sensed by an IoT device in IoT network is delivered to the IoT server through network coding.

### 3.1. Basic Concepts

#### 3.1.1. Operations at Source

A source decomposes a message to be transmitted into *b* packets. We define these packets as $P_{i},i=1,2,\cdots ,b$. The source transforms these packets into the encoded packets, $C_{j},j=1,2,\cdots ,n$, applying linear combination and transmits them to the network [22,23].

(1) $$C_{j}=\sum_{i=1}^{b}r_{ji}P_{i}$$

From Equation (1), $r_{ji}$ are composed of operations with addition and multiplication for Galois field, $GF(2^{q})$ as randomly chosen coefficients. Encoding vector, $\vec{r_{j}}=\left(r_{j1},r_{j2},\cdots ,r_{jb}\right)$ are embedded in the header of packets $C_{j}$ and this header is used for the packet reconstruction at destination [3,22,23].

#### 3.1.2. Message Recovery at Destination

When a destination receives the encoded packets (namely, the combinations), it reconstructs them using the coding coefficient. As each encoding packet is represented with a linear equation of original packets, *b*, the destination can perform the decoding mechanism to the received packets using a linear equation and recover the original message from the packets. At this time, the *b* packets which are used in each recovery are subset of the *n* packets which are generated by the source and linearly independent [22,23].

(2) $$\left(\begin{array}{cccc} r_{11} & r_{12} & \cdots & r_{1b}\\ r_{21} & r_{22} & \cdots & r_{2b}\\ & \cdots \cdots & \cdots & \\ r_{n1} & r_{n2} & \cdots & r_{nb} \end{array}\right)\left(\begin{array}{c} P_{1}\\ P_{2}\\ \vdots \\ P_{b} \end{array}\right)=\left(\begin{array}{c} C_{1}\\ C_{2}\\ \vdots \\ C_{n} \end{array}\right)$$

From Equation (2), $C_{j}$ are the encoding packets that are received at any destination node and the corresponding encoding vectors are $\vec{r_{j}}=\left(r_{j1},r_{j2},\cdots ,r_{jb}\right)$.

### 3.2. Attack Example

Let us assume an attack example in the model described in the previous section.

**Example** **1.** *Assume that a source performs mod4 operation in GF={0,1,2,3} and generates packets. The source decomposes a message into two packets, $P_{1}$,$P_{2}$, calculates a linear equation to them with redundancies, $r_{ji}$. Then it generates and transmits the combinations, $C_{1}$,$C_{2}$,$C_{3}$,$C_{4}$ as follows.*

(3) $$\left(\begin{array}{c} r_{11}P_{1}+r_{12}P_{2}=C_{1}\\ r_{21}P_{1}+r_{22}P_{2}=C_{2}\\ r_{31}P_{1}+r_{32}P_{2}=C_{3}\\ r_{41}P_{1}+r_{42}P_{2}=C_{4} \end{array}\right)$$

In transmission, packet $C_{4}$ is attacked and modified to $C_{e}$ as in Equation (4).

(4) $$r_{1}^{e}P_{1}^{*}+r_{2}^{e}P_{2}^{*}=C_{e}.$$

Since this attacked packet, $C_{e}$ seems to be a *“look-like-valid”* with a valid digital signature and a valid encrypted value, no node could recognize it until they arrived at the destination and performed the recovery operation. We assume that there are no errors due to transmission errors in the network and the destination can receive all the packets that are transmitted from the source. Eventually the destination receives all packets, $C_{1}$,$C_{2}$,$C_{3}$,$C_{e}$ as follows.

(5) $$\left(\begin{array}{c} r_{11}P_{1}+r_{12}P_{2}=C_{1}\\ r_{21}P_{1}+r_{22}P_{2}=C_{2}\\ r_{31}P_{1}+r_{32}P_{2}=C_{3}\\ r_{1}^{e}P_{1}^{*}+r_{2}^{e}P_{2}^{*}=C_{e} \end{array}\right)$$

When the destination performs the recovery operation to four packets of Equation (5), it can get six reconstruction results, as follows.

$$\left(\begin{array}{c} r_{11}P_{1}+r_{12}P_{2}=C_{1}\\ r_{21}P_{1}+r_{22}P_{2}=C_{2} \end{array}\right)\left(\begin{array}{c} r_{11}P_{1}+r_{12}P_{2}=C_{1}\\ r_{31}P_{1}+r_{32}P_{2}=C_{3} \end{array}\right)\left(\begin{array}{c} r_{21}P_{1}+r_{22}P_{2}=C_{2}\\ r_{31}P_{1}+r_{32}P_{2}=C_{3} \end{array}\right)\Rightarrow \left(P_{1},P_{2}\right)$$

$$\left(\begin{array}{c} r_{11}P_{1}+r_{12}P_{2}=C_{1}\\ r_{1}^{e}P_{1}^{*}+r_{2}^{e}P_{2}^{*}=C_{e} \end{array}\right)\Rightarrow \left(P_{1}^{\prime },P_{2}^{\prime }\right)$$

$$\left(\begin{array}{c} r_{21}P_{1}+r_{22}P_{2}=C_{2}\\ r_{1}^{e}P_{1}^{*}+r_{2}^{e}P_{2}^{*}=C_{e} \end{array}\right)\Rightarrow \left(P_{1}^{\prime \prime },P_{2}^{\prime \prime }\right)$$

$$\left(\begin{array}{c} r_{31}P_{1}+r_{32}P_{2}=C_{3}\\ r_{1}^{e}P_{1}^{*}+r_{2}^{e}P_{2}^{*}=C_{e} \end{array}\right)\Rightarrow \left(P_{1}^{\prime \prime \prime },P_{2}^{\prime \prime \prime }\right)$$

If the four packets are all valid, these six reconstructions must show all identical results. As $C_{4}$ is forged, the reconstructions including $C_{e}$ may show the incorrect results. When all reconstruction results are not identical, it means that there are errors among the received packets. However, it is still unknown which and how many packets are polluted.

Except for the reconstructed attacked packet, $C_{e}$, the other reconstructions are generated from all the correct packets and produce an identical solution. These packets can be grouped together. If all reconstruction results are not identical, we can recognize the existence of erroneous packets. If there is one attacked packet, as in Example 1, the reconstructions generated from the remaining valid packets all have the identical solution, and if the identical solution is the majority, a valid message can be found. If the reconstruction solutions include the attacked packet, $C_{e}$, it will fortunately give a different value, it is easy to apply majority rule. Although some reconstruction results including attacked packets show the identical result, majority rules can be applied if the number of these reconstructions is less than the reconstruction performed only with the correct packets. From the above example, if $\left(P_{1}^{\prime },P_{2}^{\prime }\right)\neq \left(P_{1}^{\prime \prime },P_{2}^{\prime \prime }\right)\neq \left(P_{1}^{\prime \prime \prime },P_{2}^{\prime \prime \prime }\right)\neq \left(P_{1},P_{2}\right)$, it is possible to determine $\left(P_{1},P_{2}\right)$ that is calculated from each $\left(C_{1},C_{2}\right)$, $\left(C_{1},C_{3}\right)$, and $\left(C_{2},C_{3}\right)$ combinations by applying the majority rule. However, when the number of attacked packets is much more or all of the reconstructions including attacked packets might result in one identical value, they obstruct that the destination could identify the correct reconstruction solution by applying the majority rule.

In this paper, we propose an algorithm that detects the presence of the attacked packets among the packets received at the destination and determine the valid message despite the presence of the attacked packets.

## 4. Attack Detection and Correction Algorithm Using Network Coding

In IoT architecture with network coding, the destination may not be able to reconstruct the original message correctly when any pollution exists in packets due to attacks. Sometimes destination may not be aware of the existence of attacks and can misinterpret it by reconstructing the wrong message. If the destination can detect the existence of an attack among the received packets, it could make it possible to recover the original message, even if there exists any attacked packet. Such a scheme could reduce the possibilities of retransmission due to attack and contribute to improve network efficiency.

### 4.1. Notations and Assumptions

We have some assumptions for the proposed algorithm.

- At source, a message is decomposed into *b* plain packets for transmission. Then they are transformed into *n* encoded packets with *m* redundancies using network coding.
- Any *b* packets out of the encoded *n* packets are required to recover the original message at destination.
- We assume that each packet is independently transferred with other packets.
- A malicious node can forge packets that appear to be *“look-like-valid”* combinations and send these *“look-like-valid”* packets instead of correct packets. We call it an attacked packet.
- It is assumed that there is no transmission error in the network, and all the packets transmitted by the source node are received by the destination node.

We have the following notations in this paper.

- *b*: Number of plain packets which are decomposed from a message.
- *n*: Number of combination packets which are encoded with the plain packets and redundancies. n=b+m.
- *e*: Number of attacked packets among the *n* combination packets.
- *r*: Number of non-attacked, correct packets, n=e+r.
- $\left(P_{1},P_{2},\cdots ,P_{b}\right)$: Valid original packets which are decomposed from the original message.
- $C_{i}$: Combination packets which are encoded from valid original packets and redundancies.
- $C_{j}^{e}$: Erroneous combination packets which are fabricated by any attack.
- coeff: Encoding coefficient.
- $\left\{C_{1},C_{2},\cdots ,C_{i},i>b\right\}$: a group.
- size{·}: group size, that is number of packets in a group {·}.
- expectedR{·}: Expected number of reconstructions that a group of size *i* can recover.
- actualR{·}: Actual number of reconstructions that a group of size *i* makes an identical result.

### 4.2. Characteristics of Encoded Packets

#### 4.2.1. Group

If a destination decodes the receiving packets including any attacked packets to recover the original message, more than one reconstruction result may exist. (If all received packets are valid, there will be one valid reconstruction result.) We can classify the received packets by the reconstruction solution. We classify the reconstruction according to the result, and the packets involved in generating a result are called groups. The condition of being a group is that the number of packets in the group must be greater than *b*. That is size{group}>b.

**Example** **2.** *Assume that we have encoded packets $C_{1}$, $C_{2}$, $C_{3}$, and $C_{4}^{e}$, $C_{5}^{e}$, when b=2, m=3. Here, $C_{4}^{e}$ and $C_{5}^{e}$ are attacked packets and destination could not recognize it. Let us assume that the reconstructions show the following ten results.*

$$coeff^{-1}\left(\begin{array}{c} C_{1}\\ C_{2} \end{array}\right)=\left(P_{1},P_{2}\right),\;coeff^{-1}\left(\begin{array}{c} C_{2}\\ C_{3} \end{array}\right)=\left(P_{1},P_{2}\right),\;coeff^{-1}\left(\begin{array}{c} C_{1}\\ C_{3} \end{array}\right)=\left(P_{1},P_{2}\right)$$

$$coeff^{-1}\left(\begin{array}{c} C_{1}\\ C_{4}^{e} \end{array}\right)=\left(P_{1}^{e},P_{2}^{e}\right),\;coeff^{-1}\left(\begin{array}{c} C_{2}\\ C_{4}^{e} \end{array}\right)=\left(P_{1}^{e},P_{2}^{e}\right),$$

$$coeff^{-1}\left(\begin{array}{c} C_{1}\\ C_{5}^{e} \end{array}\right)=\left(P_{1}^{e},P_{2}^{e}\right),\;coeff^{-1}\left(\begin{array}{c} C_{2}\\ C_{5}^{e} \end{array}\right)=\left(P_{1}^{e},P_{2}^{e}\right)$$

$$coeff^{-1}\left(\begin{array}{c} C_{3}\\ C_{4}^{e} \end{array}\right)=\left(P_{1}^{e^{\prime }},P_{2}^{e^{\prime }}\right),\;coeff^{-1}\left(\begin{array}{c} C_{3}\\ C_{5}^{e} \end{array}\right)=\left(P_{1}^{e^{\prime \prime }},P_{2}^{e^{\prime \prime }}\right),\;coeff^{-1}\left(\begin{array}{c} C_{4}^{e}\\ C_{5}^{e} \end{array}\right)=\left(P_{1}^{e^{\prime \prime \prime }},P_{2}^{e^{\prime \prime \prime }}\right)$$

From the above results, three reconstructions generated from $\left(C_{1},C_{2}\right)$, $\left(C_{2},C_{3}\right)$, and $\left(C_{1},C_{3}\right)$ give one identical solution, $\left(P_{1},P_{2}\right)$. So $C_{1}$, $C_{2}$, and $C_{3}$ form one group, and the group size is $size\{C_{1},C_{2},C_{3}\}=3$. Four reconstructions generated from $\left(C_{1},C_{4}^{e}\right)$, $\left(C_{2},C_{4}^{e}\right)$, $\left(C_{1},C_{5}^{e}\right)$, and $\left(C_{2},C_{5}^{e}\right)$ also give one identical solution, $\left(P_{1}^{e},P_{2}^{e}\right)$ and $C_{1}$, $C_{2}$, $C_{4}^{e}$ and $C_{5}^{e}$ form another group, and $size\{C_{1},C_{2},C_{4}^{e},C_{5}^{e}\}$ is 4. The remaining $\left(C_{3},C_{4}^{e}\right)$, $\left(C_{3},C_{5}^{e}\right)$, and $\left(C_{4}^{e},C_{5}^{e}\right)$ have solutions $\left(P_{1}^{e^{\prime }},P_{2}^{e^{\prime }}\right)$,$\left(P_{1}^{e^{\prime \prime }},P_{2}^{e^{\prime \prime }}\right)$, and $\left(P_{1}^{e^{\prime \prime \prime }},P_{2}^{e^{\prime \prime \prime }}\right)$, respectively. However $C_{3}$ and $C_{4}^{e}$ cannot form a group because they result in only one reconstruction and $size\{C_{3},C_{4}^{e}\}=2≯b$. The same applies to $\left(C_{3},C_{5}^{e}\right)$ and $\left(C_{4}^{e},C_{5}^{e}\right)$.

Suppose that *n* combinations are composed of *r* valid packets and *e* attacked packets when they arrive at a destination, n=m+b=r+e. Let the combinations that a destination receives be $\left\{C_{1},\cdots ,C_{r}\right\}\left\{C_{\left(r+1\right)}^{e},\cdots ,C_{r+e}^{e}\right\}$. The total number of possible reconstructions which are made by the received packets is nb. The number of reconstructions which are made by only valid packets is rb when r≥b. The number of reconstructions which contain at least one attacked packet, $C_{i}^{e}$, is nb−rb.

If each reconstruction which contains at least one attacked packet generates different solutions and $\left(C_{1},\cdots ,C_{r}\right)$ becomes a only group, one identical result that is recovered by the group is the only valid message. If two or more reconstructions which contain at least one attacked packet generate one identical solution, the packets which are included in these reconstructions also make a group. When we have two or more groups, we should identify a correct solution among several groups. From the above example, we should identify whether the correct solution is the first group $\left(C_{1},C_{2},C_{3}\right)$ or the second group $\left(C_{1},C_{2},C_{4}^{e},C_{5}^{e}\right)$.

#### 4.2.2. Consistency

In the previous section, a group is defined as encoded packets included in the reconstruction to recover the identical solution. This section describes how to configure attack detection and determine a valid message using group. Let *x* be the total number of encoded packets in one group and *y* be the number of the packets to be required to recover the original message. xy is the total number of reconstructions that *x* packets can generate.

When the actual number of reconstructions included in a group is identical as xy, we says that the group has consistency. The expected number of reconstructions that a group of size *x* can generate is xy, let it be expectedR{}. Let actualR{} be the actual number of reconstructions that a group of size *x* makes an identical result. If expectedR{} of a group is equal to actualR{} of this group, the group has consistency. The conditions of consistency are shown in Algorithm 1.

**Algorithm 1:** The conditions of consistency.
*if size{·}>b*

*   {·} is group*

*if expectedR{·}==actualR{·}*

*   group {·} has consistency*


From Example 2, the first group, $\left\{C_{1},C_{2},C_{3}\right\}$ has $size\{C_{1},C_{2},C_{3}\}=3$. This group has consistency because expectedR{C1,C2,C3}=32=3 and $actualR\{C_{1},C_{2},C_{3}\}$ is 3 since there are three cases of $\left(C_{1},C_{2}\right)$,$\left(C_{2},C_{3}\right)$ and $\left(C_{1},C_{3}\right)$ producing the identical solution. The size of second group, $size\{C_{1},C_{2},C_{4}^{e},C_{5}^{e}\}$ is 4, so expectedR{C1,C2,C4e,C5e}=42=6. However, $actualR\{C_{1},C_{2},C_{4}^{e},C_{5}^{e}\}$ is 4 because there are 4 cases of $\left(C_{1},C_{4}^{e}\right)$, $\left(C_{2},C_{4}^{e}\right)$, $\left(C_{1},C_{5}^{e}\right)$, and $\left(C_{2},C_{5}^{e}\right)$ producing the identical solution. Thus, this group does not have consistency since $expectedR\left\{C_{1},C_{2},C_{4}^{e},C_{5}^{e}\right\}\neq actualR\left\{C_{1},C_{2},C_{4}^{e},C_{5}^{e}\right\}$.

Assume that a group has *r* valid packets and r>b. This group has consistency because they all generate an identical result through rb reconstructions. Assume that a group has attacked packets and e>b. If the actual number of reconstruction in this group is eb and they make one identical solution, this group has also consistency.

Suppose that a group, *G* has $r^{\prime }$ valid packets and $e^{\prime }$ attacked packets. Let $r^{\prime }\geq b$ and $e^{\prime }\geq 1$. $size\left\{G\right\}=r^{\prime }+e^{\prime }$, so expectedR{G}=r′+e′b. expectedR{G}=actualR{G} must be satisfied for this group to be consistent. However, since $r^{\prime }>b$, the reconstructions produced by these valid packets are already included in rb and the actualR{G} is r′+e′b−r′b. We have actualR{G}<expectedR{G}, so this group cannot have consistency.

Consider that a group has $r^{\prime }$ valid packets and *e* attacked packets, and $r^{\prime }\leq b-1$ and $r^{\prime }+e>b$. This group can be consistent if all the reconstructions generated by the $r^{\prime }+e$ encoded packets makes the identical solution.

**Example** **3.** *Suppose that a destination receives encoded packets with b=3, m=5, n=8, r=5, and e=3, and the existence of attack packets is unknown. The encoded packets can be represented with $\left\{C_{1},C_{2},C_{3},C_{4},C_{5}\right\}\left\{C_{6}^{e},C_{7}^{e},C_{8}^{e}\right\}$. Since $\left\{C_{1},C_{2},C_{3},C_{4},C_{5}\right\}$ are valid packets, we have 53=10 reconstructions that generate an identical solution and the packets form a group, $g_{1}$ with $size\{C_{1},C_{2},C_{3},C_{4},C_{5}\}=5$. $expectedR\left\{C_{1},C_{2},C_{3},C_{4},C_{5}\right\}=actualR\left\{C_{1},C_{2},C_{3},C_{4},C_{5}\right\}=10$, so this group $g_{1}$ has consistency.*

Assume that another group, $g_{2}$ has valid packets with $r^{\prime }=3$ and an attacked packet with $e^{\prime }=1$, and include $\left\{C_{1},C_{2},C_{3}\right\}\left\{C_{6}^{e}\right\}$. Since $size\{C_{1},C_{2},C_{3},C_{6}^{e}\}=4$, $expectedR\{C_{1},C_{2},C_{3},C_{6}^{e}\}$ is 43=4. However, the reconstruction calculated by $\left\{C_{1},C_{2},C_{3}\right\}$ generates the correct solution, which is included in group, $g_{1}$. Therefore the group $g_{2}$ has actualR{C1,C2,C3,C6e}=43−1=3 and cannot have consistency.

Suppose the third group has $r^{\prime }=2$ valid packets and *e* attacked packets, and $r^{\prime }+e$ encoded packets are $\left\{C_{1},C_{2}\right\}\left\{C_{6}^{e},C_{7}^{e},C_{8}^{e}\right\}$. Suppose that reconstructions of these encoded packets generate one identical solution. $size\{C_{1},C_{2},C_{6}^{e},C_{7}^{e},C_{8}^{e}\}=5$, so $expectedR\{C_{1},C_{2},C_{6}^{e},C_{7}^{e},C_{8}^{e}\}$ is 53=10. The $actualR\{C_{1},C_{2},C_{6}^{e},C_{7}^{e},C_{8}^{e}\}$ of this group is also 10 and this group has consistency because $\left\{C_{1},C_{2}\right\}$ packets cannot generate a solution.

### 4.3. Algorithm

With group and consistency characteristics of encoded packets, we obtain the following Algorithm 2 for detecting attacks and identifying a valid message at the destination.

**Algorithm 2:** Attack detection and correction algorithm at destination.   For all *n* received encoding packets          Calculate all nb reconstructions    For all nb reconstructions          Classify and group identical reconstruction result    For all group *i*         if size{groupi}>b, set group *i* to true    For all groupi==true         if actualR{groupi}==expectedR{groupi}               set group *i* to consistency    For all groups with consistency         Find the group with the largest size

Let us consider the detailed algorithm. First let us discuss the conditions under which valid reconstruction exists. When the reconstruction forms a consistent group, the message generated by this group are candidate for valid reconstruction. When e<m, we have r>b and rb>1. These r valid packets participate in two or more reconstructions to generate the identical solution and form a consistent group. Therefore the condition that there exists a valid solution is e<m. Even if the other reconstructions, nb−rb form a group with one identical solution, the group will not be consistent. This is because the size of this group is *n* and expectedR{thisgroup}=nb>actualR{thisgroup}=nb−rb.

**Example** **4.** *Suppose that encoded packets with b = 2, m = 2, and n = 4 are transmitted and a destination receives these packets with an attacked packet with e = 1. This example is the case of m>e, so the condition that a valid reconstruction exists is satisfied. Since there are valid packets with r=3, the destination can get rb = 3 reconstructions, which is both actualR and expectedR of this group, and verify the consistency of this group. The other reconstructions are 42−32 = 3. Even if we get an identical result, $size\{C_{1},C_{2},C_{3},C_{4}^{e}\}$ = 4 and $expectedR\{C_{1},C_{2},C_{3},C_{4}^{e}\}$ = 6 makes it inconsistent. Thus, we can find the valid solution group.*

If the destination receives packets with e=2 and r=2, only one valid reconstruction can be obtained through r=2, so it is not possible to determine if it is a valid message. In this case, the condition of m>e is not satisfied.

Next, consider the conditions under which majority rule can be applied to find a group that generates a valid message when there are multiple consistent groups. We have two conditions as follows.

1. Consider the case where r>e(r≥b,e≥b). Two groups $g_{r}$ with group size *r* and $g_{e}$ with group size *e* can be formed, and the expectedR of each group becomes rb and eb. We have $expected\left\{g_{r}\right\}>expected\left\{g_{e}\right\}$, so it is possible to apply majority rule to identify the valid message.
2. Suppose that a group $g_{r}\prime$ consists of $\left\{C_{1},\cdots ,C_{r^{\prime }}\right\}\left\{C_{1}^{e},\cdots ,C_{e}^{e}\right\}$ packets, and $r^{\prime }\leq b-1$ and $r^{\prime }+e>b$. Assume that the reconstructions of $r^{\prime }+e$ encoded packets generate one identical result. Since $r^{\prime }\leq b-1$, $r^{\prime }$ encoded packets could not generate a solution. If $r^{\prime }+e<r$, expected{gr′}=r′+eb<rb, and this group $g_{r}\prime$ is not subject to majority rule and the group $g_{r}$ can be selected by majority rule. For this group to be consistent, the maximum value of $r^{\prime }$ would be b−1. Now let be $r^{\prime }=b-1$. With $r^{\prime }+e<r$ and n=b+m=r+e, we have $e\leq \lfloor \frac{m}{2}\rfloor$. When $e\leq \lfloor \frac{m}{2}\rfloor$, even if this group with attacked packets has one identical solution and it is consistent, $expectedR\left\{g_{r}\prime \right\}<expectedR\left\{g_{r}\right\}$, therefore the group $g_{r}\prime$ is dropped by majority rule.

**Example** **5.** *A destination that receives encoded packets with b=3, m=5, n=8, e=4, and r=4 are represented with $\left\{C_{1},C_{2},C_{3},C_{4}\right\}\left\{C_{5}^{e},C_{6}^{e},C_{7}^{e},C_{8}^{e}\right\}$. Here we have m>e, r=e, and $e>\lfloor \frac{m}{2}\rfloor$, these satisfy the condition that a valid recovery exists, but do not satisfy the conditions (1) and (2) to find a valid recovery. Let us consider the detail. From valid packet group $\left\{C_{1},C_{2},C_{3},C_{4}\right\}$ with $size\{C_{1},C_{2},C_{3},C_{4}\}=4$, we have expectedR{C1,C2,C3,C4}=actualR{C1,C2,C3,C4}=rb=4. If four attacked packets generate one identical solution, group $\left\{C_{5}^{e},C_{6}^{e},C_{7}^{e},C_{8}^{e}\right\}$ with $size\{C_{5}^{e},C_{6}^{e},C_{7}^{e},C_{8}^{e}\}=4$, would have $actualR\{C_{5}^{e},C_{6}^{e},C_{7}^{e},C_{8}^{e}\}=4$ which is the same as expectedR{C5e,C6e,C7e,C8e}=eb=4 and has consistency. The r valid packet group is consistent, and the e attacked packet group is also consistent. Then we will not be able to identify the correct solution.*

From the above considerations, we have conditions, e<m, r>e, and $e\leq \lfloor \frac{m}{2}\rfloor$. Finally, the conditions for identifying a valid solution is r>e and $e\leq \lfloor \frac{m}{2}\rfloor$. In the first case of Example 4, since e<m, r>e, and $e\leq \lfloor \frac{m}{2}\rfloor$ with b=2, m=2, r=3, and e=1, we can see that the condition to find a valid message is satisfied.

## 5. Performance Analysis and Results

### 5.1. Performance Analysis

Suppose that the probability that an encoded packet is attacked in transmission is *p*. The probability that the number of attacked packets *e* is equal to *i* is as follows.

(6) P(e=i)=nipi(1−p)n−i.

Let us consider all the conditions to find a valid message. The probability that $e\leq \lfloor \frac{m}{2}\rfloor$ is,

(7) P(e≤⌊m2⌋)=∑i=0⌊m2⌋nipi(1−p)n−i.

We have the probability that e<r,

(8) P(e<r)=∑j=0r−1njpj(1−p)n−j.

The probability to find a valid message $P_{g}$ is equal to the probability that $e\leq \lfloor \frac{m}{2}\rfloor$ and e<r, and can be expressed as shown in Equation (9),

(9) Pg=∑k=0min(⌊m2⌋,r−1)nkpk(1−p)n−k.

Here, we define a cost function related to the transmission proportional to the number of transmitted packets. Assume that the cost of transmitting one packet is $C_{tx}$. If the message is successfully recovered at the destination with probability of $P_{g}$, the cost is $n\cdot C_{tx}$. We have $1-P_{g}$ probability that a message cannot be successfully recovered at its destination, in which case the message will be re-transmitted until it is successfully recovered. Therefore, the average number of attempts to deliver a message to its destination is calculated as $\frac{1}{P_{g}}$, and the total cost can be expressed as

(10) TotalCost=1Pg·n·Ctx=n·Ctx∑k=0min(⌊m2⌋,r−1)nkpk(1−p)n−k.

### 5.2. Results and Discussion

**Figure 2:**
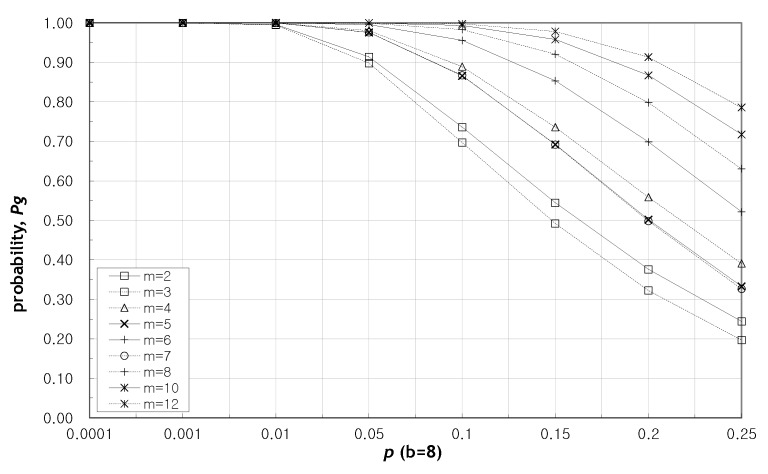
The probability of finding a valid solution as the probability *p* increases when b=8.

Figure 2 shows that as the probability of a packet being attacked increases, the probability of finding a valid message decreases. Also, as the redundancy increases, the probability $P_{g}$ also increases. Looking at the example of b=4, m=4 in the graph, it can be seen that according to Equation (9), the value of *e* becomes the minimum value between $\left\lfloor \frac{m}{2}\right\rfloor$ and maximum value of r−1 with e<r, so that even if two attacked packets occur, the recovery result can be found with high probability. Here we see that the probability of identifying a valid message, $P_{g}$ when redundancy *m* is even, is higher than when *m* is odd. The reason is as follows. Let $m_{1}$ be an even denoted by $m_{1}=2q,\;\;q=1,2,\cdots$. We have $\left\lfloor \frac{m_{1}}{2}\right\rfloor =\left\lfloor \frac{2q}{2}\right\rfloor =q$, and $n_{1}=b+2q$. Now, let $m_{2}$ be an odd denoted by $m_{2}=2q+1,\;\;q=1,2,\cdots$. We have $\left\lfloor \frac{m_{2}}{2}\right\rfloor =\left\lfloor \frac{2q+1}{2}\right\rfloor =q$, and $n_{2}=b+2q+1$. Then, $n_{1}<n_{2}$, but $\left\lfloor \frac{m_{1}}{2}\right\rfloor =\left\lfloor \frac{m_{2}}{2}\right\rfloor =q$. In Equation (9), *k* has the identical value in both $n_{1}$ and $n_{2}$ even though $n_{2}>n_{1}$. Then we have ${\left(1-p\right)}^{\left(n_{1}-q\right)}>{\left(1-p\right)}^{\left(n_{2}-q\right)}$. Therefore, the probability $P_{g}$ shows lower when m=2q+1,q=1,2,⋯. However, since (1−p)≃1 when *p* is small enough, the probability $P_{g}$ is not affected by this characteristic of redundancy.

**Figure 3:**
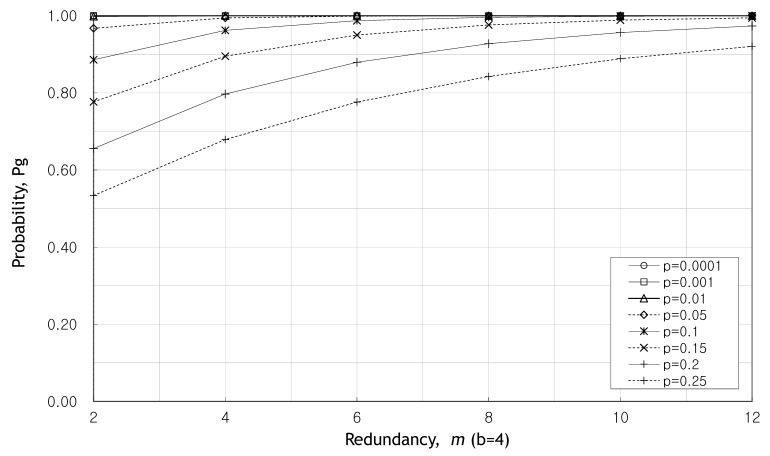
$P_{g}$ as the redundancy, *m*, increases when b=4.

**Figure 4:**
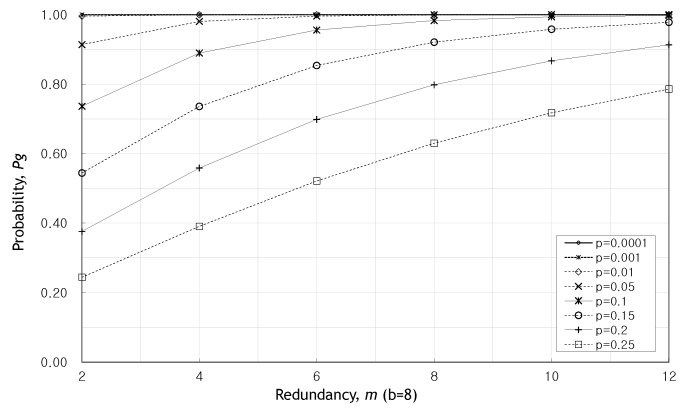
$P_{g}$ as the redundancy, *m*, increases when b=8.

We look into the probability $P_{g}$ for the case where *m* is even in Figure 3 and Figure 4. Figure 3 and Figure 4 show the change of the probability $P_{g}$ as the redundancy *m* increases when b=4 and b=8. In these figures, the greater the redundancy, the higher the probability $P_{g}$. The probability $P_{g}$ does not have been affected by *p* as redundancy gets larger. Hence the probability $P_{g}$ is stabilized at m=8.

**Figure 5:**
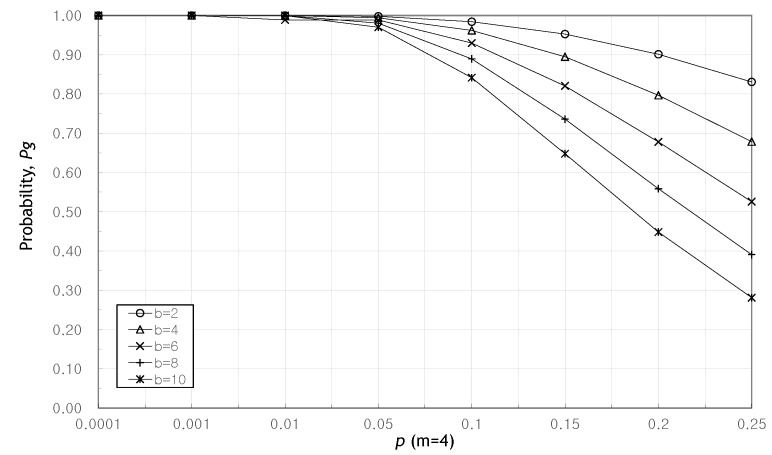
$P_{g}$ as the number of packets increases when m=4.

Figure 5 shows the change in probability $P_{g}$ with increasing *p* when m=6. We can see that the probability $P_{g}$ remains almost one until p=0.05 regardless of the value of *b*. It can be seen that when the number of source packets, *b* is larger, $P_{g}$ decreases rapidly due to the increase in *p*. Figure 2, Figure 3, Figure 4 and Figure 5, fewer packets and greater redundancies give higher probability of obtaining a valid message at the destination.

**Figure 6:**
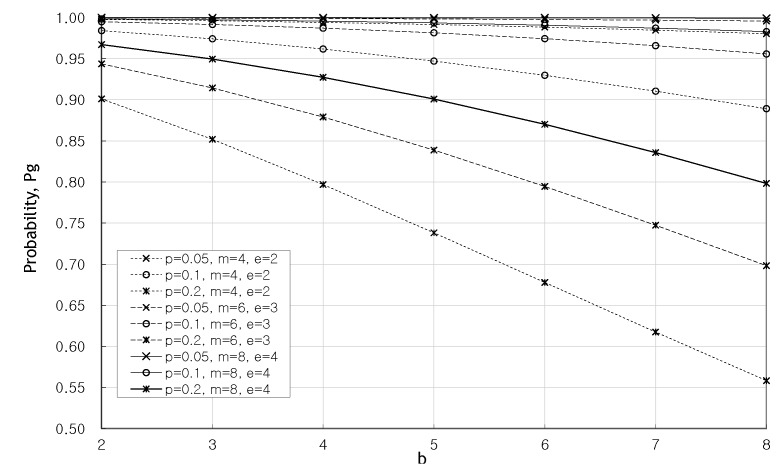
$P_{g}$ as *b* increases when e=2,3,4.

Figure 6 shows the change in the probability $P_{g}$ of recovering a valid message as *b* increases when 2, 3, and 4 attacked packets occur, respectively. It can be seen that *m* must be increased to cope with the high *p* value and the increase of the attacked packet *e*. Despite the high probability of p=0.2 at b=8, $P_{g}=0.558$ at m=4, and $P_{g}=0.798$ at m=8, and we have a much higher recovery probability $P_{g}$ at m=8.

**Figure 7:**
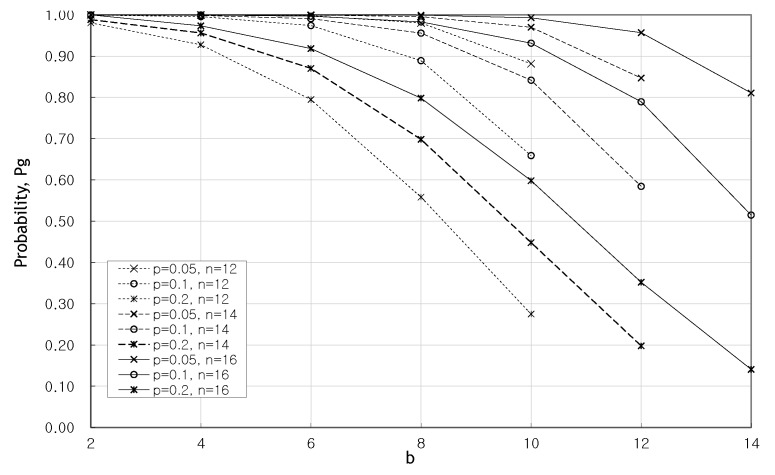
$P_{g}$ with the change of *b* and *m* when fixing *n*.

Figure 7 shows the probability $P_{g}$ with the change of *b* and *m* when fixing *n*. Even with a high probability of p=0.2, if *b* maintains more than half of *n*, it can be seen that a recovery probability of 0.8 or more can be obtained. In the case of b=10, when m=6 and p=0.1, a higher recovery probability is obtained in spite of higher *p* than when n=12 and p=0.05.

**Figure 8:**
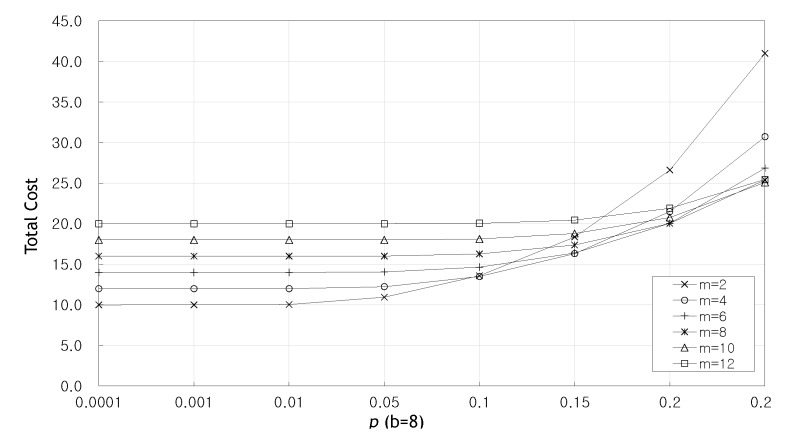
Total cost as the probability *p* increases when b=4.

Figure 8 shows the total cost as the probability *p* increases when b=8. Until p=0.1, it can be seen that the total cost remains stable regardless of the value of *m*. Increasing the value of *m* increases the total number of packets *n*, thus affecting the transmission cost, while increasing the probability $P_{g}$ so that the total cost can be stabilized.

**Figure 9:**
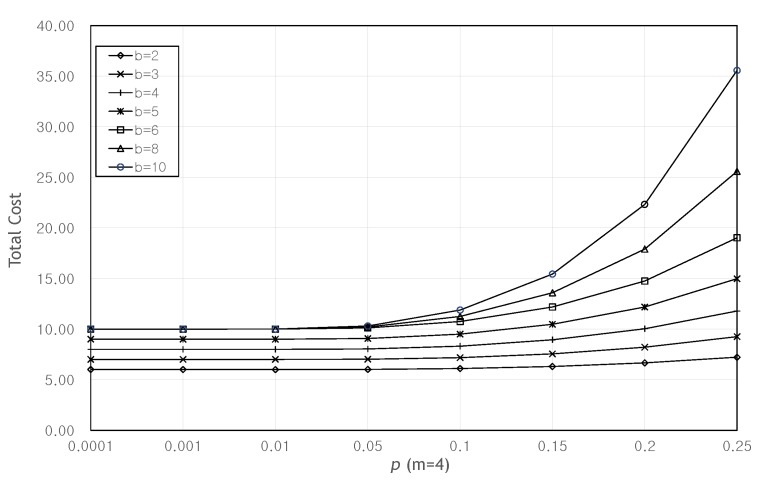
Total cost as the probability *p* increases when m=4.

Figure 9 show the total cost as the number of original packets *b* and the probability *p* increase when m=4. This graph also shows that until p=0.1, the total cost remains stable regardless of the value of *b*. From Figure 8 and Figure 9, we can see that when p≥0.1, the larger *m* and the larger *b*, the larger $P_{g}$, so the total cost can be reduced.

The study to ensure the security of network coding discussed in chapter 2 includes the method of applying cryptographic techniques such as encryption algorithms and digital signatures in [1,4,8,9,10,11], the method of secure network coding proposed in [7,9,17], and the prevention of attacks by distinguishing the trusted nodes in [13,15]. In the method of applying a cryptographic technique, there is overhead such as key exchange, and if an internal attacker modifies the packet and encrypts it with a correct key, the destination decrypts it and trusts the packet and executes the recovery for network coding. In this process, the destination cannot recognize the forgery of the packet. Secure network coding is also aimed at preventing the occurrence of polluted packets and cannot cope if polluted packets occur. In the method based on trust, internal attacks by trusted nodes cannot be prevented. While the existing methods focus on preventing the occurrence of attacked packets, the proposed method can be a solution that can detect the presence of attack and recover messages even if a packet is attacked by an internal attack.

## 6. Conclusions

Network coding has contributed to improving the throughput of a network in IoT environment. In the network coding in which the intermediate node of the routing combines and encodes the packets received from the neighboring nodes, and transmits the combinations, it is inevitable that a combination of forged or corrupted packets by the malicious node occurs. It also allows for the presence of *“look-like-valid”* attacked packets that appear to have valid signatures and valid encryption by malicious nodes that hide their identity.

We focused on the case where a destination does not recognize the existence of this kind of attacked packets. When the destination recovers a message with packets containing the attacked packets, all recovery results may not match and the destination will get the wrong result. This paper has proposed an algorithm that detects the presence of attacked packets among received packets and can identify a valid message even if attacked packets exist. We also analyzed the conditions under which the proposed algorithm can operate. This algorithm has shown that even with a high probability of attack, a valid message can be identified with a high probability. The results showed a high recovery probability when the number of redundancies is about half of the total packets even under high attack probability. Even if attacks occur, the message can be recovered without re-transmitting the packet, which shows that the recovery cost gradually increases even with the increase of redundancy or attack.
